# Recruiting to an infant formula-milk feeding trial: lessons from the baby milk trial

**DOI:** 10.1186/1745-6215-16-S2-P121

**Published:** 2015-11-16

**Authors:** F Whittle, K K Ong, S J Griffin, R Lakshman

**Affiliations:** 1 University of Cambridge, Cambridge, UK

## Background

The Baby Milk Trial is a RCT of a multi-component intervention to prevent excessive milk intakes and weight gain in formula-fed infants. Healthy term infants receiving formula-milk are recruited up to 3 months old. There are both practical and social barriers to engaging this population, particularly the perceived stigma of formula-feeding from both the community and health professionals. We describe the recruitment methods employed in this study.

## Method

A multi-level approach is used to identify formula-feeding parents through: Research Staff on a Postnatal Hospital ward; health professionals, including GPs, Midwives and Health Visitors; and via a mail-out using the NHS integrated database “Systm1”. Eligible families are invited to attend recruitment visits at local research facilities or offered home visits if required.

## Results

Over 650 infants have been recruited, identified through: GP Surgeries, 41%; Systm1 mail-out, 28%; Research Staff on postnatal wards, 23%; Health Visitors, 1%; Midwives 1%; Other (including posters and word of mouth), 6%. Having agreed to take part, more than 1/3 of mothers asked for home visits rather than receive travel costs to attend local research facilities.

## Conclusions

To identify formula-feeding families within 3 months of birth, GPs, research staff and a mail-out from a routine NHS database were effective strategies. Home visits reduce the burden on participants and facilitate recruitment and retention. Future research studies in this population should consider how to better engage with Health Visitors and Midwives who have the most frequent contacts with these families.

